# Letters from Far Japan

**Published:** 1891-12-12

**Authors:** Ernest Hart


### Letters from Far Japan.

By Mrs. Ernest Hart.

### 1.—A JAPANESE PRISON.

That "stone walls do not a prison make" is characteristically true of prisons in Japan. It would be strange if a people so poetic and unique as the Japanese did not treat crime and criminals in a way different from any other nation, and inasmuch as their morality, religion, and standards differ from ours, so do their prisons.

Among many experiences, I can count none more gloomy and dispiriting than a visit paid to an English model prison The grey blank walls, the cold cheerless cells, the solitary prisoners at work at useless tasks, the exercise ground with the men making their daily dreary rounds, the low repulsive faces of the criminals, and the stolid indifference of the warder with his bunch of clanking keys, all combine to leave on the mind a weary sense of some of the hopeless results of advanced civilisation.

A visit to a Japanese prison leaves quite other memories. The people who can laugh at a funeral, and who are rarely seen to cry (except at the theatre), are bound to look at crime not too seriously, and to treat criminals more as those who have gone a little astray than as persons radically bad. "Do go and see a prison before you leave Japan," said a lady friend to me; "you will be so interested; it is one of the prettiest things I have seen in Japan." So I went. The prison I visited was the large State prison situated in the outskirts of the great spreading city of Tokio. Drawn by two fast runners yoked in the jinrikisha, or small hand car, which takes the place of cab or carrsage in Japan, we sped along at the rate of about seven miles an hour, through the busy streets of Tokio, across the broad river, and out into the suburbs, where the lilac blooms of the westeria, and the flame-coloured masses of flowers of the azaleas were making all the gardens gay.

On arriving at the prison premises, I was struck at once by the fact that there were no outside walls, and that the gates which led into the large garden and farm which surround the buildings stood wide open. The prison contained, however, 1,661 prisoners, 1,542 men and 119 women, and of this number 1,062 men and 51 women had been committed for robbery.

Accompanied by the officials, and by a Japanese friend who spoke English fluently and who acted as interpreter, I visited all the workshops, dormitories and infirmaries of the large establishment. The prison consists of a series of disconnected workshops, sheds, and buildings. They are all built of wood, and are so constructed that air circulates freely through them; the most scrupulous cleanliness is observed everywhere, and every care is taken to secure proper sanitation. The plan of the prison in separate unconnected buildings which cover a large area of ground, and their perfect ventilation due to their method of construction, must make sanitation much simpler matter than it becomes in a prison solidly constructed and situated in the narrower limits of a city.

The dress of the prisoners consists of a coral-coloured kimono, which is a long, loose garment folded across the chest and confined at the waist by a sash; and a blue kerchief worn round the neck. Industrial work of all kinds is carried on by the prisoners in large open sheds. In one the metal hairpins worn by women are being hammered out; in another coarse cloth is being woven; and picking and teazing cotton, grinding rice, coopering, dyeing, making mats, and carpentering are the occupations in many separate workshops. Several men and boys are employed at a small factory for making the Japanese sauce, soy. The workshops are bright, clean, and airy, and the busy workmen, dressed in their picturesque pink kimonos, give to the whole an impression of prison life and discipline different to any received before from visits made to the prisons of other nations. Two warders are considered sufficient to keep watch over forty prisoners, who are said to be docile and obedient, and to make no attempt to escape. The hours of labour are from eight to ten a day. If a prisoner works diligently he is commended and given a ribbon to wear, and he may also have his term of imprisonment shortened. If, on the other hand, he is idle, his food rations are diminished till he sees the wisdom of industry. When he has been confined more than one hundred days, he can begin to earn something for himself by his labour. The officials state that they find no difficulty in obtaining a market for the prisoners' work. In some prisons a large amount of cloisonné enamels is produced. Everywhere the effort is made to make the term of imprisonment of educational benefit to the prisoner, and to turn the criminal into a good artisan.

In the reformatory department of the Tokio prison there were 194 boys under sixteen years of age, who had been chiefly committed for robbery and incendiarism. They attend school in the morning, and work in the factory in the afternoon. Among these boys were twenty-three who were distinguished from the others by being dressed in blue. These had been committed when under twelve years of age, and could now leave if they were fortunate enough to have friends or relatives to take them out, but who would otherwise be left to be brought up with the prison as their only home. I pitied these poor homeless lads, and think that the ladies of Japan would do well to take them under their protection and provide them with home and work outside the prison gates.

In one large empty shed were seen seven men who patiently and silently sat on their heels on the matted floor before a warden who kept silence as he watched. These were prisoners condemned for a short term of imprisonment without hard labour; but the silence and enforced inactivity are felt to be so severe a strain, that after two or three days, these prisoners generally beg for and obtain the privilege of hard labour, or rather the pleasant occupation of the workshops.

The dormitories are large, lofty sheds with matted floors, and with wooden walls made of narrow vertical lattices through which the air passes freely. In the winter these open walls can be closed with wooden shutters. Each mat, one yard wide, serves as a bed for two men, the covering being a padded quilt. Nothing could be more healthy and cleanly than this form of dormitory, and it conforms at the same time with the simple habits of the Japanese, the most wealthy of whom sleep on the floor, covered with straw matting, the bed being formed of a padded "futon," or quilt, a second futon taking the place of bedclothes.

The infirmary is constructed on the same principle as the dormitories. Close by are ovens and appliances for disinfecting clothes, &c. Large hot baths are provided and are used daily by the prisoners. Offences against prison discipline are punished by confinement in the solitary cells where the prisoners are put on low diet and set to pick hemp. These solitary cells were well lit little buildings with open bars, standing apart in grounds. The real dark cell is similarly constructed, but is said to be very rarely used for serious offences against prison rules. In the garden stands another building which I must mention, namely the execution shed. There are on an average about two executions a year. The shed stands on the confines of the large well-cultivated vegetable garden, and on the day I saw it, no signs were visible of the dismal tragedies enacted there from time to time. In fact it is not possible to imagine a less repulsive execution shed. Just outside, the flowers are blooming, the birds singing, and the sun shining, and the gates which lead to liberty are standing wide open. It must be hard indeed to part with life at that place and moment, and to take the plunge into the great unknown, and we are glad to learn that in this "land of gentle manners" murders and executions are not matters of of daily occurrence. The officials whose duty it is to see the last act performed are so placed that they only see the dead body as it drops, and not the preparations made for hanging.

On the women's side of the prison, weaving is the principal occupation. As I passed through the long shed and noted the low degradation, the repulsive expression, and the hateful ugliness which crime could give to the pretty child-like faces of Japanese women, I was struck by the beautiful face of one of the prisoners, who, with her pink kimono wrapped closely round her graceful figure, applied herself the more diligently to her loom when she found she was observed. "Who is that beautiful woman," I asked, "and why is she here?" I was told she was once a well-known Jeisha, or dancing girl, called Hai Ume, and that, inspired by hatred of a servant whom she suspected of making quarrels between herself and her parents, she stabbed and killed him. She is undergoing imprisonment for life—a sad and pitiful example of wasted gifts and opportunities. Five of the women prisoners had been committed for murder, of which it was said the passion of love was the motive.

